# To the Editors of the Medical and Physical Journal

**Published:** 1809-09

**Authors:** 


### *To the Editors of the Medical and Physical Journal.*

Gentlemen,

I Believe there is but one danger or one difficulty attendant exclusively on the *operation* of Lithotomy, (viz.) the probability of the beak of the Gorget slipping from the groove of the staff.

To obviate this inconvenience, therefore, is the object of the present paper; but as my contrivance for this end is simple, and the attainment of it important, I am inclined to suspect that the same has been attempted, and laid aside, from a decided conviction of its impracticability. If, however, such should not have been the case, and your superior judgment detect no palpable imperfection in the plan, I should be obliged by the insertion of it in your Journal.

I would have the beak of the gorget made larger than usual, and the neck belonging to the beak (if I may so express myself) rather thinner; the front edge of it might be made sharp, so as to divide the urethra with the more ease; thus much for the gorget.

I would have a certain portion in the groove of the staff, (viz.) somewhat about an inch on each side the curvature of it, almost closed up by a gradual approximation of its sides; so that in its practical application, when the beak of the gorget arrives at the bend of the staff, which is both the critical and *contracted* part, the beak being larger than the slit of the staff in the place, and the neck of the beak being smaller; the gorget must follow the course of the staff, so long as it cannot slip out of it, and the contraction of the groove may be continued to within an inch of its extremity; thus may the gorget be extricated from the staff with the ordinary facility.

The excellence of a communication is not in proportion to the pages it occupies; I shall therefore briefly conclude, by observing, that as a pupil, I am not in a situation for proving the excellence of my plan by the test of experiment; but many are in such a situation, to whom the contents of your Journal are familiar. To such I apply; let them lay the credit of the invention to the proposer, and reserve the honour of its first application to themselves.

*Birmingham, June 21,* 1809.

